# P-1303. Traditional 6 months Vs Shorter Duration Treatment Regimen for Drug Susceptible Tuberculosis: A Systematic Review and Meta-Analysis with Subgroup Analysis

**DOI:** 10.1093/ofid/ofaf695.1491

**Published:** 2026-01-11

**Authors:** Ashesh Das, Naga Sai Akhil Reddy Gogula, Muhammad Shahzaib, Urvashi Bharia, Vanshika Chaudhary, Ananya Talukdar, Dhruvi Dipakkumar Pathak, Debvarsha Mandal, Anika Goel, Divyanshu Kumar, Muhammad Roshaan, Jana Al Jnainati, Harsh Sangwan

**Affiliations:** KPC Medical College and Hospital , Kolkata, India, Kolkata, West Bengal, India; Duke University, Cary, North Carolina; King Edward Medical University Lahore, sialkot, Punjab, Pakistan; Lokmanya Tilak Municipal Medical College and General Hospital, Mumbai, Navi Mumbai, Maharashtra, India; Maharishi markandeshwar institute of medical sciences and research, Karnal, Haryana, India; Bharati Vidyapeeth Medical College, Pune, India, Delhi, Delhi, India; Government medical College surat, Surat, Gujarat, India; Avalon University School of Medicine, Cypress, TX; Kakatiya medical College, Telangana, Warangal, Telangana, India; Medical College, Kolkata, Cleveland, Ohio; King Edward Medical University, Lahore, Punjab, Pakistan; University of Milano-Bicocca, Bergamo, Lombardia, Italy; NAMO Medical Education and Research institute, silvassa, Dadra and Nagar Haveli, India

## Abstract

**Background:**

Despite the recent advances in TB, the standard 6-month regimen for drug-susceptible TB poses challenges both in adherance and development of drug resistance. Shorter regimens may be a similarly effective alternative with improved compliance and reduced toxicity. This Systematic Review and Meta-Analysis evaluates the efficacy, safety, and completion rates of such regimens versus traditional six months therapy.

**Methods:**

A systematic search of PubMed, Embase, Scopus, and Cochrane Library identified Randomized Controlled Trials (RCTs) comparing 6 months regimen with shorter duration regimens in drug susceptible TB through March 2025. Data were analyzed using RevMan 4.2.1. Pooled risk ratios (RRs) with 95% confidence intervals (CIs) were calculated using Mantel-Haenszel methods. Random- or fixed-effects models were applied based on heterogeneity (Higgins’ I²). Statistical significance was set at p < 0.05. Risk of bias was assessed using RoB 2.0.

**Results:**

Fourteen RCTs (N = 10,555) were included. Shorter or novel regimens significantly reduced unfavorable outcomes (RR = 1.07; 95% CI: 0.90–1.26; p = 0.04) and relapse (RR = 2.35; 95% CI: 1.17–4.70; p = 0.02). No significant difference was observed in sputum culture conversion at 2 months (RR = 1.05; p = 0.48; I² = 96%), treatment completion (RR = 1.00; p = 0.77; I² = 61%), mortality (RR = 0.90; p = 0.60; I² = 0%), loss to follow-up (RR = 0.88; p = 0.39; I² = 57%), recurrence (RR = 1.53; p = 0.15; I² = 73%), or adverse events (RR = 1.07; p = 0.45; I² = 77%). Subgroup analyses also showed variable heterogeneity across outcomes.

**Conclusion:**

The Short novel regimens may reduce relapse and unfavorable outcomes without increasing mortality but there is high heterogeneity. Observed heterogeneity is likely due to variation in treatment duration, drug regimens, variations in control groups and local antibiotic resistance. Findings support shorter regimens as viable alternatives, but further head-to-head trials are needed to bring a change in the global clinical practice.

**Disclosures:**

All Authors: No reported disclosures

